# Oolong tea attenuates neuroinflammation by modulating the microbiota-gut-brain axis in a rat model of autism

**DOI:** 10.3389/fnut.2025.1643147

**Published:** 2025-09-10

**Authors:** Peng Zheng, Hongbo Zhao, Xingliang Zhang, Qiuting Wu, Zhen Zheng, Shaoqun Liu

**Affiliations:** ^1^College of Horticulture, South China Agricultural University, Guangzhou, China; ^2^Department of Respiratory Medicine, Institute of Pediatrics, Affiliated Shenzhen Children’s Hospital of Shantou University Medical College, Shenzhen, China; ^3^Department of Pediatrics, The Third Affiliated Hospital, Sun Yat-sen University, Guangzhou, China

**Keywords:** autism, oolong tea, microbiota-gut-brain axis, neuroinflammation, TLR-4/IκB-α/NF-κB

## Abstract

**Background:**

Autism spectrum disorder (ASD) is a prevalent neurodevelopmental disorder with limited effective treatments. Emerging evidence implicates dysregulation of the microbiota-gut-brain axis in ASD pathogenesis. Oolong tea (OT), a traditional Chinese tea with neuroprotective properties, may modulate this axis, but its effects and mechanisms in ASD remain unclear. We investigated whether OT attenuates neuroinflammation in a valproic acid (VPA)-induced rat model of autism through the microbiota-gut-brain axis and the TLR-4/IκB-α/NF-κB signaling pathway.

**Methods:**

An ASD model was established by prenatal VPA exposure (500 mg/kg, i.p., E12.5). Postnatal VPA-treated rats received OT (100, 200, or 400 mg/kg/day) for 4 weeks. Behavioral assessments included self-grooming, marble burying, and three-chamber social interaction tests. Nissl staining evaluated neuropathology. Gut microbiota composition was analyzed using 16S rRNA sequencing of fecal samples. Lipopolysaccharide (LPS), interleukin-6 (IL-6), and tumor necrosis factor-α (TNF-α) levels were measured in the plasma, intestine, and brain using enzyme-linked immunosorbent assay. Intestinal and blood-brain barrier (BBB) integrity (claudin-1/5, occludin, ZO-1) and TLR-4/IκB-α/NF-κB pathway activation were assessed by Western blot/immunofluorescence. Microglial (Iba-1) and astrocytic (GFAP) activation and neuronal TLR-4 localization (co-staining with Neun) were examined. Antibiotic cocktail (ABX)-induced microbiota depletion validated gut microbiota dependency.

**Results:**

OT (400 mg/kg/day) significantly ameliorated repetitive behaviors (reduced self-grooming duration and marble burying), sociability deficits (improved sociability/social preference index), and attenuated cortical neuronal loss in VPA-treated rats. OT restored gut microbiota dysbiosis, specifically reducing pathogenic *Ruminococcaceae* and *Bacteroides* abundances. It decreased LPS, IL-6, and TNF-α levels in the plasma, intestine, and cortex, while enhancing intestinal and BBB tight junction protein expression. OT suppressed TLR-4/IκB-α/NF-κB activation in both intestine and cortex, with TLR-4 predominantly localized to neurons, and reduced microglial/astrocytic activation. Critically, ABX treatment abolished OT’s neuroprotective effects and restored neuroinflammation.

**Conclusion:**

OT attenuates ASD-like phenotypes and neuroinflammation in VPA-treated rats by rebalancing gut microbiota, restoring intestinal/BBB barriers, and inhibiting neuronal TLR-4/IκB-α/NF-κB signaling. This study highlights OT’s potential as a microbiota-targeted therapeutic strategy for ASD.

## 1 Introduction

Autism spectrum disorder (ASD) is a prevalent neurodevelopmental disorder characterized by deficits in social interaction and communication, alongside restricted interests and repetitive behaviors. It affects approximately 1 in 36 children in the United States (1). ASD imposes a significant lifelong burden on individuals, families, and society, posing major public health challenges. Critically, the fundamental mechanisms underlying ASD remain incompletely understood, and effective, specific pharmacological treatments are currently lacking. Consequently, identifying novel therapeutic strategies for ASD is of paramount importance.

Emerging evidence implicates dysregulation of the microbiota-gut-brain axis in the pathogenesis of neurological disorders, including Alzheimer’s disease (2), Parkinson’s disease (3), and depression (4). This bidirectional communication network, crucially mediated by the gut microbiota, regulates brain development and behavioral function (5, 6). Distinct gut microbiota profiles have been identified in individuals with ASD compared to neurotypical children (7, 8). Furthermore, fecal transplantation from ASD donors to germ-free mice has been shown to induce ASD-like behaviors and alter microbial communities (9). These findings strongly suggest that gut microbiota dysbiosis plays a causal role in ASD etiology and brain dysfunction, highlighting microbiota-targeted therapies as a promising novel treatment modality.

Oolong tea (OT), a traditional Chinese tea, possesses neuroprotective properties attributed to its anti-inflammatory (10), antioxidant (11), and mitophagy promotion effects (12). Notably, Song et al. (13) recently demonstrated that OT attenuates cognitive impairment induced by circadian rhythm disruption through the modulation of the microbiota-gut-brain axis. However, it remains unexplored whether OT exerts neuroprotective effects against ASD and what specific role the microbiota-gut-brain axis plays in mediating these potential effects.

Valproic acid (VPA), a clinical antiepileptic and histone deacetylase (HDAC) inhibitor, is a well-established agent for inducing rodent models of ASD (14, 15). While VPA directly impacts neuronal development through HDAC inhibition (16), growing evidence underscores its significant impact on the gastrointestinal tract, which contributes to ASD-like pathogenesis (17, 18). VPA disrupts gut microbiota homeostasis (19, 20), downregulates intestinal tight junction proteins (e.g., claudin-1, occludin, ZO-1) (21), increases intestinal permeability, and alters short-chain fatty acid production (22). These changes promote the proliferation of Gram-negative bacteria, which are recognized for high lipopolysaccharide (LPS) production (23). The resulting intestinal barrier dysfunction and proinflammatory microbiota community facilitate LPS translocation into systemic circulation. LPS subsequently compromises the blood-brain barrier (BBB), enabling proinflammatory factors to infiltrate the brain, activate neuroinflammatory pathways, and ultimately contribute to ASD-like behaviors.

The causal role of microbiota-derived changes in ASD-like behaviors is supported by preclinical evidence. Systemic LPS injection in rodents recapitulates core autistic phenotypes (24). Furthermore, fecal microbiota transplantation from VPA-treated rodents transfers both gut dysbiosis and ASD-like behaviors to germ-free mice (25). This positions the VPA model as ideal for investigating microbiota-gut-brain axis targeted therapies like OT.

A key pathway implicated in this gut-brain communication involves the LPS activation of toll-like receptor-4 (TLR-4), which leads to the degradation of the inhibitor of κB-α (IκB-α), nuclear translocation of nuclear factor kappa B (NF-κB), and subsequent transcription of proinflammatory cytokines, including interleukin-6 (IL-6), tumor necrosis factor-α (TNF-α), and interleukin-1β (IL-1β) (26, 27). BBB impairment allows circulating LPS and cytokines to enter the brain, promoting neuroinflammation (28). It remains unknown whether OT modulates this TLR-4/IκB-α/NF-κB pathway in the context of ASD.

Therefore, this study aimed to determine whether OT ameliorates ASD-like behaviors and neuronal damage in VPA-treated rats, evaluate whether OT modifies gut microbiota composition, assess whether OT decreases systemic and central LPS levels and inflammation, investigate whether OT improves intestinal and BBB integrity and modulates the TLR-4/IκB-α/NF-κB pathway in a cell-type-specific manner, and finally validate the dependence of OT’s effects on gut microbiota using antibiotic-induced microbiota depletion. By addressing these objectives, we sought to provide mechanistic insights into OT’s potential as a microbiota-gut-brain axis modulator for ASD intervention.

## 2 Materials and methods

### 2.1 Animals

The experiments were approved by the Animal Use and Care Committee at South China Agricultural University (Approval No. 2021B144). A total of 30 specific pathogen-free 12-week-old Sprague–Dawley rats (20 female rats, 10 male rats, 250–300 g) were acquired from Guangdong Medical Laboratory Animal Center. Rats were housed under controlled conditions (temperature: 23 ± 1 °C, humidity: 55 ± 5%, 12 h light/dark cycle) with ad libitum access to standard chow and sterile water for 1 week of acclimatization before experiments. Female and male rats were mated at a 2:1 ratio. Embryonic day 0.5 (E0.5) was designated as the day of vaginal plug detection, while birth was considered postnatal day (PND) 0.

On E12.5, pregnant rats in the VPA group received 500 mg/kg VPA (Sigma, P4543; 250 mg/mL saline solution) by intraperitoneal injection, while sham group rats were injected with an equivalent volume of saline.

### 2.2 OT extraction, component analysis, and supplementation

The tea extracts were prepared as follows. First, 10 g of the OT sample was soaked in 100 mL of boiling water for 10 min, after which it was extracted three times. All the extracts were subsequently mixed, filtered, and concentrated under vacuum. Finally, the concentrated filtrate of tea extracts was freeze-dried into powder and stored at −80 °C.

Component analysis of OT was performed according to a previous study (29). For caffeine, 0.45 g magnesium oxide and 0.1 g freeze-dried tea powder were added to 30 mL of ultrapure water at 100 °C and ultrasonically agitated for 30 min. Then, 1 mL of the extract was filtered and analyzed using high-performance liquid chromatography (HPLC). The analysis utilized a C18 SB column (4.6 × 250 mm, 5 mm) at 35 ± 1 °C with 100% ultrapure water as solvent A and 100% methanol as solvent B. The compounds were eluted for 14 min at an isocratic pressure of 70% A and 30% B at a flow rate of 0.9 mL/min with a detection wavelength of 280 nm.

For theanine, 0.1 g freeze-dried tea powder was extracted in 10 mL of ultrapure water at 100 °C for 30 min. Then, 1 mL of the extract was filtered and analyzed using HPLC. The mobile phases were 100% ultrapure water (A) and 100% acetonitrile (B). The gradient was set at 100% A for the first 12 min, gradually shifted to 20% A from 12 to 14 min, and held at 20% for 5 min. Finally, the gradient was changed to 100% A from 19 to 21 min and then was held for 4 min. Theanine was detected at 210 nm.

For catechins, 0.2 g freeze-dried tea powder was extracted in 8 mL of 70% methanol. Then, 1 mL of supernatant was filtered and analyzed by HPLC using a C18 column (4.6 × 250 mm, 5 mm). The catechin monomer was eluted using a gradient elution procedure with 0.1% aqueous formic acid (v/v) (A) and 100% acetonitrile (B) as the mobile phases. The gradient elution started at 8% B for 5 min, was increased to 25% from 5 to 14 min, and decreased to 8% from 14 to 30 min. Detection was at 280 nm.

Rats were randomly assigned to six groups with 12 rats in each group. In the sham group, the sham rats received distilled water (400 mg/kg/day) through gavage for 4 weeks (from PND21 to PND49). In the VPA group, VPA-treated rats were administered distilled water (400 mg/kg/day) through gavage during this period. In the OT-L, OT-M, and OT-H groups, VPA-treated rats were administered three doses of OT, viz. 100 mg/kg/day, 200 mg/kg/day, and 400 mg/kg/day through gavage for 4 weeks (from PND21 to PND49), respectively (30–32). In the OT + antibiotic cocktail (ABX) group, VPA-treated rats were administered OT (400 mg/kg/day) through gavage and with antibiotics (ampicillin 1 g/L, vancomycin 0.25 g/L, neomycin 1 g/L, and metronidazole 1 g/L) added into their drinking water for 4 weeks (from PND21 to PND49) (33).

### 2.3 Behavioral tests

Based on preliminary data, a minimum sample size of six animals per group was required to detect significant behavioral differences (34). Six to eight rats per group were used and analyzed. Animals were randomly assigned to groups, and investigators remained blinded to group assignments throughout the study and analysis. Behavioral testings were conducted at fixed daily times to avoid circadian effects.

Exclusion criteria: Rats were excluded if they died during the experiment, showed >20% weight loss, or failed to complete behavioral tests (e.g., persistent immobility or aggressive behavior).

#### 2.3.1 Self-grooming test

At PND45, a self-grooming test was conducted. The self-grooming test was used to assess rodent stereotyped behavior (35). The spontaneous grooming behavior was calculated in this test as previously described (36). The rat was moved into an empty cage. The behaviors of the rats were observed for 10 min to record the total duration of self-grooming. Self-grooming behaviors were defined as rubbing the head, face, nose, and ears, as well as licking the body, anus, genitals, and tail. The observer sat approximately 1.5 m from the rat test cage.

#### 2.3.2 Marble burying test

At PND46, a marble burying test was carried out as described previously (37). Repetitive digging was assessed using the marble burying test. Each rat was placed into a cage (48 × 35 × 20 cm) with a new bedding approximately 5 cm deep. Twenty marbles, sized 4 × 5, were placed on the bedding surface. Subsequently, the rat was positioned at the cage’s central point and allowed a 30-min period of free exploration. After 30 min of undisturbed exploration (observer absent), marbles with at least 70% burial depth in the bedding were calculated.

#### 2.3.3 Three-chamber social interaction test

At PND48, a three-chamber social interaction equipment was used to study social behavior in rats. The three-chamber social interaction test was performed as previously described (38). The test device was a transparent plexiglass container with dimensions of 60 cm × 60 cm × 40 cm, which was split into three compartments. Each compartment was linked by a 10 cm × 10 cm opening. Between different trials, the three-compartment container was wiped down with 70% ethanol, dried using paper towels, and then left to dry naturally in the air. To help the rats get used to the surrounding environment, they were moved into the test device 1 h before the test. Each rat was put into the middle compartment alone and given 5 min to explore without restriction.

During the sociability assessment phase, a stranger rat was caged and positioned in one side chamber, while an empty cage was positioned in the opposing chamber. The test rat was then introduced into the central chamber and permitted to probe the side chambers for a duration of 10 min. The stranger rats were age- and sex-matched to the test rats. The time spent by the test rat in either side chamber was recorded.

During the social preference phase, the rat that was originally regarded as a stranger is now considered familiar. Another unfamiliar rat was positioned within the empty cage. The unfamiliar rats were age- and sex-matched to the test rats. Therefore, the chambers are then a familiar chamber and an unfamiliar chamber. The amount of time the experimental rat spent in either side chamber was recorded. After each experiment, the chambers and doorways were sanitized with 70% ethanol.

The following formulas were used to compute the sociability index and social preference index:


$$\begin{array}{l} \text{Sociability index}=\text{Cumulative time in the stranger chamber}\\ /\text{Cumulative time in the empty chamber} \end{array}$$

$$\begin{array}{l} \text{Social preference index}=\text{Cumulative time in unfamiliar chamber}\\ /\text{Cumulative time in familiar chamber} \end{array}$$

### 2.4 Microbiota analysis

At PND49, fresh feces samples were collected. The 16S rRNA gene amplicon sequencing and analysis were performed by OE Biotech Co., Ltd. (Shanghai, China). Total genomic DNA was isolated using a DNA Extraction Kit. Quality and quantity of DNA were assessed using NanoDrop and agarose gel. The diluted DNA served as a template for polymerase chain reaction amplification of bacterial 16S rRNA genes, with the barcoded primers and Takara Ex Taq (Takara) employed in the reaction. For analyzing bacterial diversity, V3–V4 variable regions of 16S rRNA genes were amplified using universal primers 343F and 798R.

Raw sequencing data were in FASTQ format. Paired-end reads underwent preprocessing with the cutadapt software to identify and remove adapter sequences. After trimming, paired-end reads were subjected to filtering of low-quality sequences, denoising, merging, and chimera detection and removal using DADA2 with QIIME2 (2020.11). At last, the software outputs the representative reads and the amplicon sequence variant (ASV) abundance table. The representative read of each ASV was selected using the QIIME2 package. All representative reads were annotated and blasted against Silva138_Eukaryota^1^ using q2-feature-classifier with the default parameters.

All bioinformatics analyses of amplicon reads were conducted based on ASVs. α diversity was computed using R software (version 3.5.1). Principal coordinates analysis (PCoA) based on weighted UniFrac distances was visualized with the ggplot2 package (version 3.4.0) in R software. LEfSe, a tool commonly applied to identify biomarkers, is capable of uncovering metagenomic features (39). Sequence data have been deposited in the NCBI SRA database (Accession Number: PRJNA1298520).

### 2.5 Blood and tissue collection

The rats were euthanized at PND50. The plasma samples were obtained for enzyme-linked immunosorbent assay (ELISA) analysis. The intestine and brain were stored at −80 °C and used for Western blot analysis. The intestine and brain were placed in 4% paraformaldehyde, followed by Nissl staining and immunofluorescence analysis.

### 2.6 Nissl staining

Nissl staining was employed to identify neuronal damage. Paraffin sections were cleared three times (10 min each), with excess liquid gently shaken off between steps. They were then dehydrated through an ethanol series (anhydrous, 95, 85, 75%; 5 min each), rinsed in distilled water (3 times), and stained with preheated 1% toluidine blue (50 °C, then 56 °C for 20 min). After thorough distilled water washes, sections were differentiated under microscopic control (using 95% ethanol or 0.1% glacial acetic acid) until Nissl bodies were distinct. Rapid dehydration in anhydrous ethanol was followed by clearing and mounting with neutral balsam. Photos of cortical neurons were taken with a conventional optical microscope to view their morphology.

### 2.7 ELISA

The expression levels of lipopolysaccharide (LPS), interleukin-6 (IL-6), and tumor necrosis factor-α (TNF-α) in the plasma, intestine, cortex, and hippocampus were evaluated using rat ELISA kits (Ek-Bioscience, Shanghai, China). Before initiating the assay, all reagents were prepared. Wells for standards and test samples were designated; 50 μL of the standard was dispensed into each standard well. For test sample wells, 10 μL of the sample was added, followed by 40 μL of sample diluent. Then, 100 μL of HRP-conjugate reagent was added to every well, and the plate was sealed with an adhesive cover and incubated at 37 °C for 60 min. After incubation, the contents of each well were aspirated, and the wells were washed. This process was repeated four times, for a total of five washes. Following the final wash, any residual wash solution was removed by aspiration or decantation. The plate was then inverted and blotted on clean paper towels. Next, 50 μL of chromogen solution A and 50 μL of chromogen solution B were added to each well. The plate was mixed gently and incubated at 37 °C for 15 min. Subsequently, 50 μL of stop solution was added to each well. Within 15 min, the optical density was measured at 450 nm using a microplate reader.

### 2.8 Immunofluorescence analysis

Initially, sections were dewaxed for 10 min. They were then rinsed for 5 min each in anhydrous ethanol, 95% ethanol, and 75% ethanol. The slices were then placed in an EDTA antigen repair solution and boiled. After being removed from the retrieval solution, the sections were immersed in TBST three times. Next, 10% donkey serum was added and incubated at 37 °C for 30 min. The sections were then incubated with ZO-1 antibody (1:50, Thermo Fisher, 61-7300), TLR-4 antibody (1:200, Proteintech, 19811-1-AP), Neun (1:500, Oasis Biofarm, OB-PGP006-01), Iba-1 antibody (1:200, Oasis Biofarm, OB-PGP049-01), and GFAP antibody (1:200, Oasis Biofarm, OB-PGP055) overnight at 4 °C. The next day, the sections were rinsed three times with TBST. A 100 μL aliquot of the secondary antibody working solution was added to each section and incubated at 37 °C for 45 min. Subsequently, 100 μL of the DAPI working solution was added to each section to stain the nuclei. After a 5-min light exposure, the sections were rinsed with TBST. Finally, the sections were visualized under a fluorescence microscope.

### 2.9 Western blot

The Western blot analysis was performed according to previously reported procedures (40). Total protein content from the intestine and cortex of the rat was extracted using ristocetin-induced platelet aggregation buffer supplemented with protease inhibitors. Nuclear protein and cytoplasmic protein were extracted using a nuclear and cytoplasmic extraction kit (Thermo Fisher, 78833). Protein concentrations were quantified using the BCA protein concentration kit (Beyotime, P0010S). The protein underwent electrophoresis using a 10% SDS-PAGE gel and was subsequently transferred to a 0.45 mm PVDF membrane. The membrane was then incubated in a protein blocking solution for 1 h. The membranes underwent incubation with the following primary antibodies: claudin-1 (1:250, Invitrogen, 51-9000), occludin (1:1,000, CST, 91131), ZO-1 (1:250, Invitrogen, 61-7300), claudin-5 (1:250, Sigma, SAB4502981), TLR-4 (1:500, Invitrogen, PA5-23124), IκB-α (1:1,000, CST, 9242), NF-κB (1:1,000, CST, 8242), GAPDH (1:1,000, CST, 2118s), and Histone H3 (1:1,000, ab1791). On the following day, the membranes were rinsed three times with TBST solution, then incubated with a second antibody (1:5,000, Boster, BA1054) for 1 h at room temperature. After incubation, the membrane was washed three times with TBST solution to eliminate unbound secondary antibodies. Finally, the protein bands were observed using enhanced chemiluminescence. The protein expression levels were quantified using ImageJ software.

### 2.10 Statistical analysis

Data analysis was performed using SPSS 17.0 software. The results are expressed as the mean ± standard error of the mean (SEM). Statistical evaluations were carried out using one-way analysis of variance, followed by Fisher’s *post hoc* test for pairwise comparisons. A *p*-value threshold of <0.05 was adopted to define statistical significance.

## 3 Results

### 3.1 OT has neuroprotective effects on VPA-treated rats

**Figure 1:**
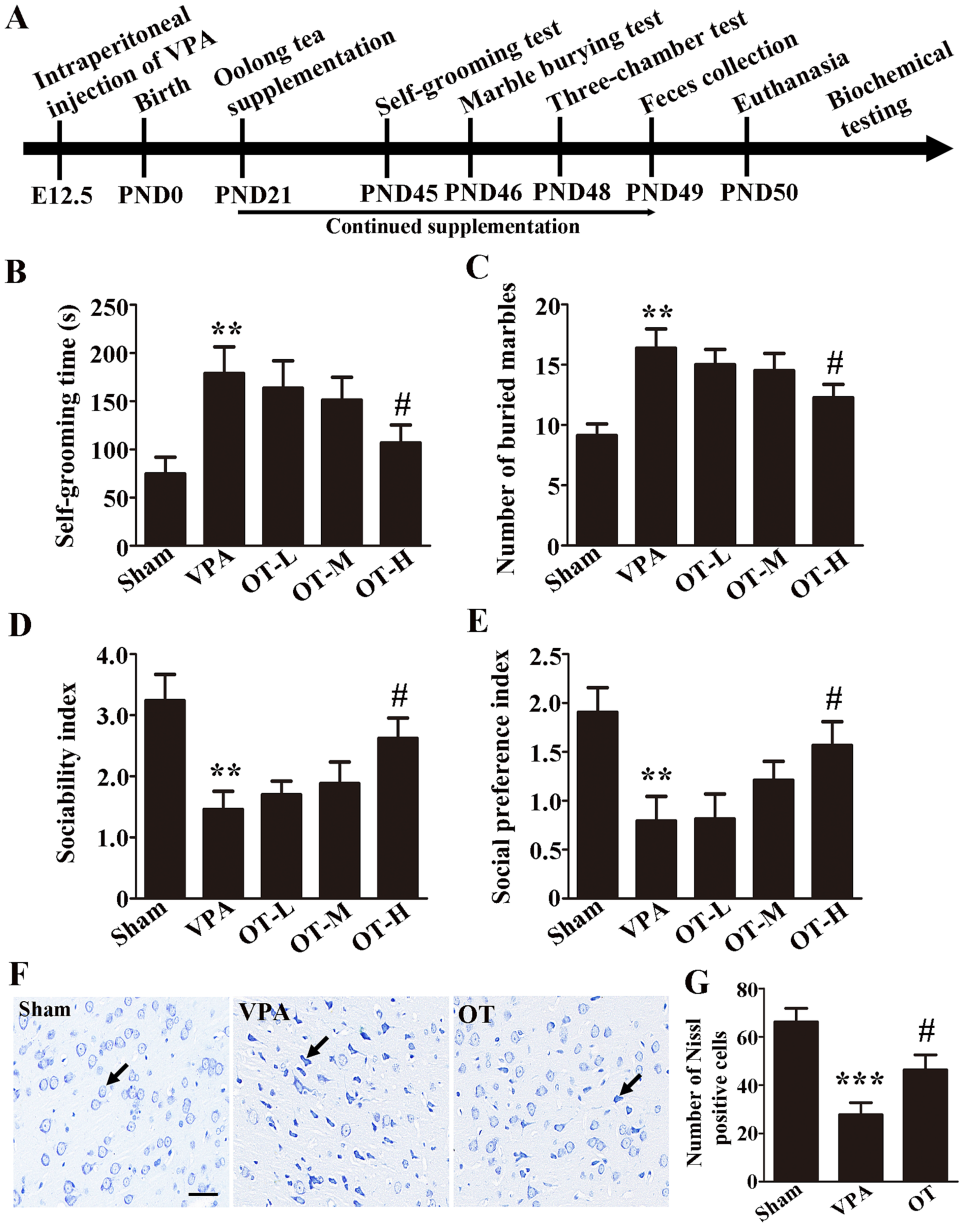
OT has neuroprotective effects on VPA-treated rats. **(A)** Experimental timeline. **(B)** Self-grooming time; *n* = 6/group. **(C)** Number of marbles buried in the marble burying test; *n* = 8/group. **(D)** Sociability index; *n* = 6/group. **(E)** Social preference index; *n* = 6/group. **(F)** Nissl staining. **(G)** Quantification of Nissl-stained cells in the cortex. The data are expressed as the mean ± SEM, ^**^p < 0.01 and ^***^p < 0.001 for the VPA group versus the sham group, ^#^*p* < 0.05 for the OT group versus the VPA group.

The experimental timeline is displayed in Figure 1A. VPA was administered at E12.5, and OT was orally administered from PND21 to PND49. The self-grooming test was conducted on PND45, the marble burying test was measured on PND46, the three-chamber test was performed on PND48, feces were collected on PND49, and the rats were euthanized on PND50.

The contents of caffeine, theanine, and catechins in the extract of OT are shown in Table 1.

**Table 1:** Contents of caffeine, theanine, and catechins in the extract of OT.

Tea component	Oolong tea (mg/g)
Catechin (C)	4.09 ± 0.04
Epicatechin (EC)	7.00 ± 0.25
Epicatechin gallate (ECG)	24.85 ± 0.27
Epigallocatechin (EGC)	35.75 ± 2.46
Epigallocatechin gallate (EGCG)	79.02 ± 0.30
Gallocatechin (GC)	7.65 ± 1.21
Gallocatechin gallate (GCG)	23.68 ± 0.31
Caffeine	70.62 ± 0.26
Theanine	14.80 ± 0.08

To evaluate the neuroprotective effects of OT on VPA-treated rats, behavioral tests were carried out. To assess repetitive behaviors in rats, the self-grooming test and marble burying test are frequently utilized. In the self-grooming test, the VPA group spent significantly more time self-grooming than the sham group (Figure 1B, *p* < 0.01). In contrast, the OT-H group exhibited shorter self-grooming time than the VPA group (Figure 1B, *p* < 0.05). No statistically significant differences were observed in the self-grooming test for the OT-L group and the OT-M group compared to the VPA group (Figure 1B, *p* > 0.05).

Marble burying test indicated that the VPA group buried more marbles than the sham group (Figure 1C, *p* < 0.01). The OT-H group buried fewer marbles than the VPA group (Figure 1C, *p* < 0.05). No statistically significant differences were observed in the marble burying test for the OT-L group and the OT-M group compared to the VPA group (Figure 1C, *p* > 0.05).

The three-chamber social interaction test was used to assess sociability and social preference. As shown in Figures 1D,E, the VPA group exhibited a lower sociability index and social preference index than the sham group (Figures 1D,E, *p* < 0.01), whereas OT-H supplementation reversed the results (Figures 1D,E, *p* < 0.05). No statistically significant differences were observed in the sociability index and social preference index for the OT-L group and the OT-M group compared to the VPA group (Figures 1D,E, *p* > 0.05). Therefore, we administered 400 mg/kg/day of OT to VPA-treated rats for all subsequent assays.

Moreover, Nissl staining was used to assess the neuroprotective effects of OT on VPA-treated rats. The VPA group showed significantly fewer Nissl-stained cells in the cortex than the sham group (Figure 1G, *p* < 0.001). However, OT supplementation significantly attenuated the neuronal cell death in the OT group compared with the VPA group (Figure 1G, *p* < 0.05). Taken together, these findings suggest that OT has neuroprotective effects on VPA-treated rats.

### 3.2 OT modifies the microbiota composition in VPA-treated rats

**Figure 2:**
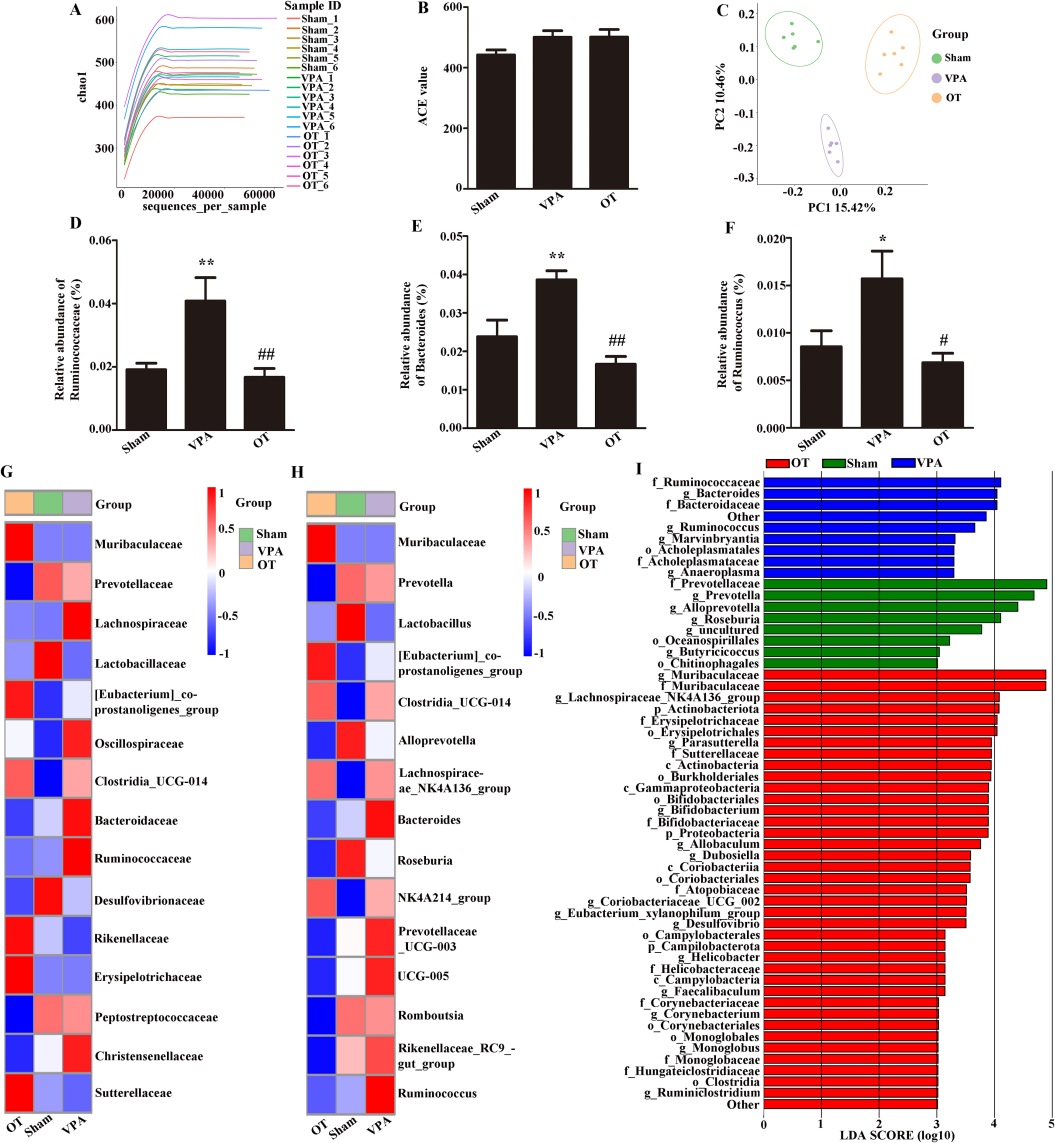
OT modifies gut microbiota composition in VPA-treated rats. **(A)** Chao1 index. **(B)** ACE index. **(C)** PCoA chart. **(D–F)** Comparison of the relative abundances of the *Ruminococcaceae*, *Bacteroides*, and *Ruminococcus* in the three groups. **(G)** Heatmap analysis of the gut microbiota in different groups at the family level. **(H)** Heatmap analysis of the gut microbiota in different groups at the genus level. **(I)** Analysis of different gut microbiota of the three groups using LEfSe software. The data are expressed as the mean ± SEM; *n* = 6/group. ^*^p < 0.05, and ^**^p < 0.01 for the VPA group versus the sham group; ^#^*p* < 0.05, and ^##^*p* < 0.01 for the OT group versus the VPA group.

To characterize the gut microbiota in VPA-treated rats, fecal samples were subjected to 16S rRNA gene sequencing analysis. The Chao1 index indicates sample richness (Figure 2A). The analysis of α diversity using the abundance-based coverage (ACE) estimator revealed no significant differences (Figure 2B, *p* > 0.05), indicating comparable gut microbiota diversity across the three groups. Conversely, PCoA revealed significant variations in gut microbiota composition among the groups (Figure 2C, *p* = 0.001). Moreover, compared with the sham group, the VPA group showed a marked increase in the family *Ruminococcaceae* and the genera *Bacteroides* and *Ruminococcus* (Figures 2D–F, *p* < 0.05 or *p* < 0.01). Furthermore, the OT group had lower abundances of these gut microbiota than the VPA group (Figures 2D–F, *p* < 0.05 or *p* < 0.01).

At the family level, heatmap analysis (Figure 2G) showed that the VPA group had a higher abundance of *Ruminococcaceae* than the sham and OT groups. At the genus level, heatmap analysis (Figure 2H) showed that the VPA group had a higher abundance of *Bacteroides* and *Ruminococcus* than the sham and OT groups.

LEfSe is a new way to identify biomarkers through category comparisons. The gut microbiota exhibiting the most substantial differences (with an LDA score >3.0) among the three groups were detected (Figure 2I). *Ruminococcaceae*, *Bacteroides*, *Bacteroidaceae*, *Ruminococcus*, *Marvinbryantia*, *Acholeplasmatales*, *Acholeplasmataceae*, and *Anaeroplasma* were more abundant in the VPA group. Taken together, these findings imply that OT has the potential to alter the microbiota composition in the intestines of VPA-treated rats.

### 3.3 OT decreases LPS and inflammation in VPA-treated rats

**Figure 3:**
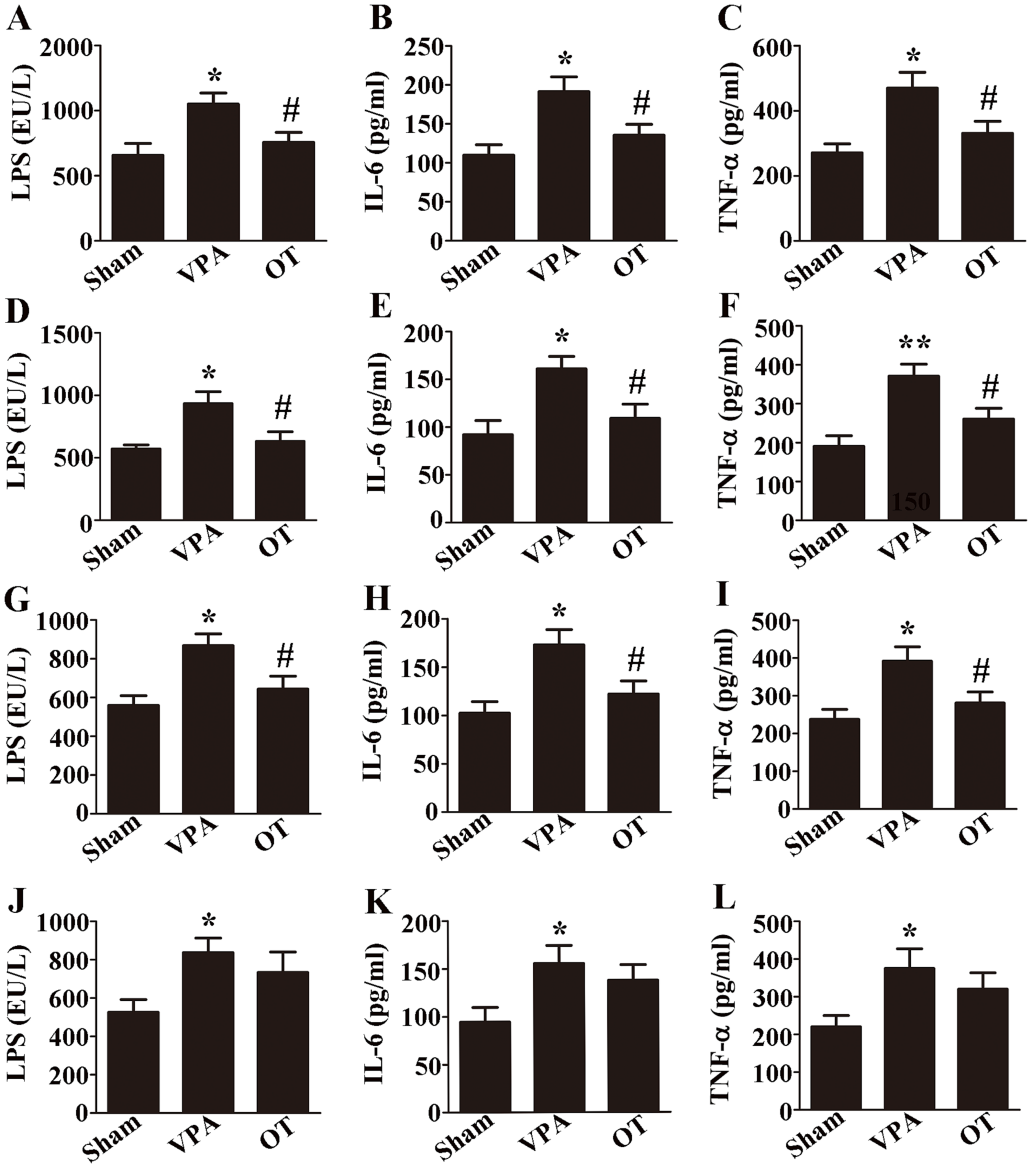
OT decreases LPS and inflammation in VPA-treated rats. **(A–C)** Plasma levels of LPS, IL-6, and TNF-α. **(D–F)** Intestinal levels of LPS, IL-6, and TNF-α. **(G–I)** Cortical levels of LPS, IL-6, and TNF-α. **(J–L)** Hippocampal levels of LPS, IL-6, and TNF-α. The data are expressed as the mean ± SEM. *n* = 3/group. ^*^p < 0.05, and ^**^p < 0.01 for the VPA group versus the sham group; ^#^*p* < 0.05 for the OT group versus the VPA group.

To investigate whether OT affects LPS and proinflammatory cytokines in VPA-treated rats. ELISA was employed to assess the levels of LPS, IL-6, and TNF-α in the plasma, intestine, cortex, and hippocampus. ELISA revealed that compared with the sham groups, the VPA group exhibited significantly elevated levels of LPS, IL-6, and TNF-α in the plasma (Figures 3A–C, *p* < 0.05), intestine (Figures 3D–F, *p* < 0.05 or *p* < 0.01), cortex (Figures 3G–I, *p* < 0.05), and hippocampus (Figures 3J–L, *p* < 0.05). Specifically, OT supplementation significantly reduced these levels in the plasma (Figures 3A–C, *p* < 0.05), intestine (Figures 3D–F, *p* < 0.05), and cortex (Figures 3G–I, *p* < 0.05) in the VPA-treated rats. There were no statistically significant differences in the levels of LPS, IL-6, and TNF-α in the hippocampus between the VPA group and the OT group (Figures 3J–L, *p* > 0.05). Therefore, we focused on the cortex in the following assays. These results indicate that OT inhibits LPS production and alleviates inflammation in VPA-treated rats.

### 3.4 OT attenuates intestinal barrier dysfunction and inhibits TLR-4/IκB-α/NF-κB signaling pathway in the intestines of VPA-treated rats

**Figure 4:**
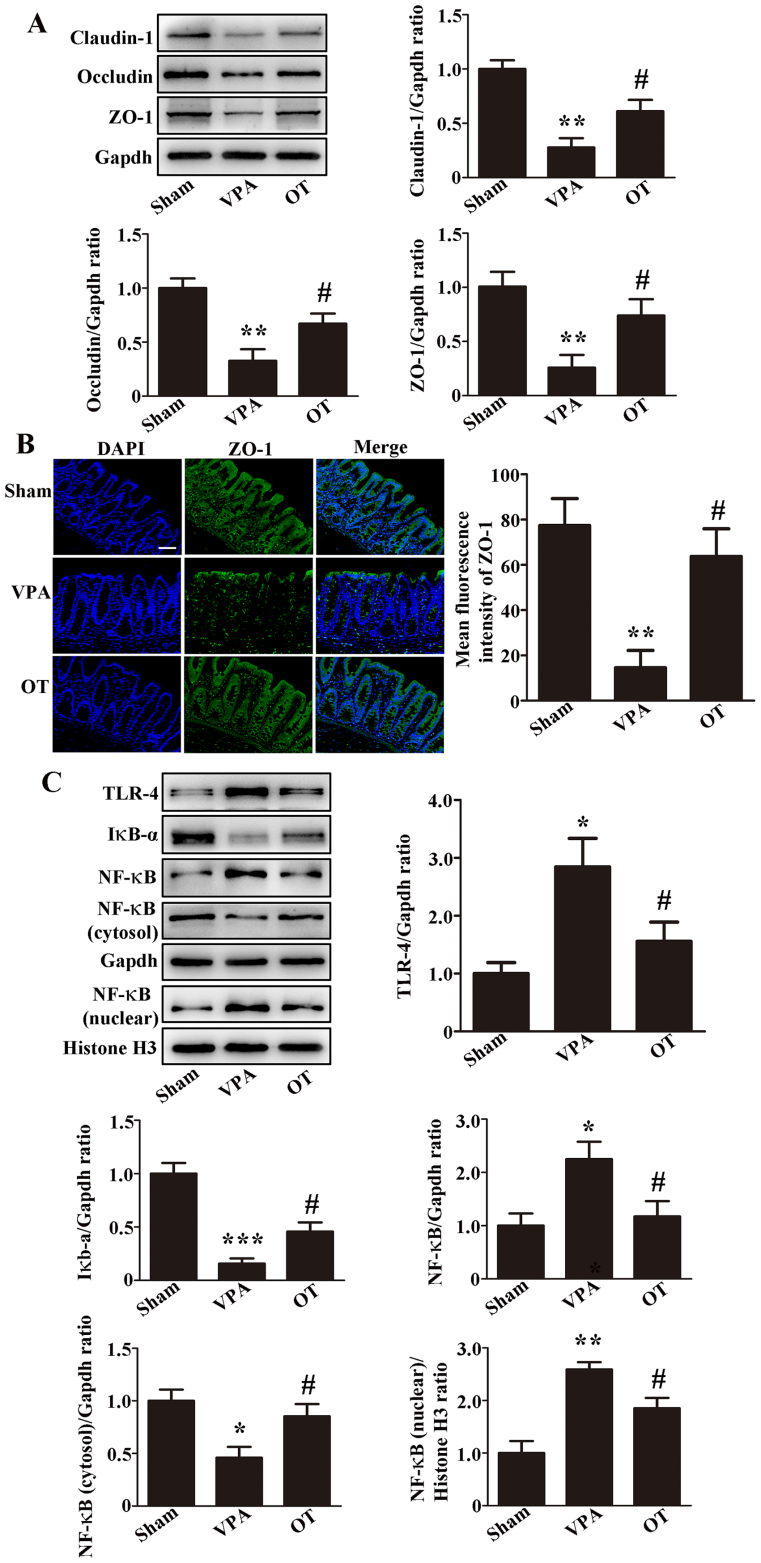
OT attenuates intestinal barrier dysfunction and inhibits the TLR-4/IκB-α/NF-κB signaling pathway in the intestines of VPA-treated rats. **(A)** Representative images and quantification of claudin-1, occludin, and ZO-1 expression in the intestine. The levels were normalized to GAPDH. **(B)** Representative images and quantification of ZO-1-positive cells in the intestine. **(C)** Representative images and quantification of TLR-4, IκB-α, NF-κB, NF-κB (cytosol), and NF-κB (nuclear) expression in the intestine. The levels of TLR-4, IκB-α, NF-κB, and NF-κB (cytosol) were normalized to GAPDH. The level of NF-κB (nuclear) was normalized to Histone H3. The data are expressed as the mean ± SEM. *n* = 3/group. ^*^p < 0.05, ^**^p < 0.01, and ^***^p < 0.001 for the VPA group versus the sham group; ^#^*p* < 0.05 for the OT group versus the VPA group.

To investigate intestinal barrier dysfunction in VPA-treated rats, Western blotting was employed to analyze tight junction proteins in the intestine. In comparison to the sham group, the VPA group exhibited notably reduced expression of the intestinal claudin-1, occludin, and ZO-1 (Figure 4A, *p* < 0.01). In contrast, the OT group demonstrated significantly elevated expression of these proteins relative to the VPA group (Figure 4A, *p* < 0.05). Immunofluorescence staining further revealed that the OT group had more intestinal ZO-1-positive cells than the VPA group (Figure 4B, *p* < 0.05). Collectively, these findings suggest that OT attenuates intestinal barrier dysfunction.

We next examined the association between OT and TLR-4/IκB-α/NF-κB signaling pathway in the intestines of VPA-treated rats. In comparison to the sham group, the VPA group exhibited upregulated expression of the intestinal TLR-4, NF-κB, and NF-κB (nuclear), with significant downregulation of IκB-α and NF-κB (cytosol) (Figure 4C, *p* < 0.05, *p* < 0.01, or *p* < 0.001). In contrast, the OT group showed downregulated TLR-4, NF-κB, and NF-κB (nuclear) expression, accompanied by upregulated IκB-α and NF-κB (cytosol) expression, compared to the VPA group (Figure 4C, *p* < 0.05). Collectively, these findings imply a potential role for the TLR-4 signaling pathway in the protective effect of OT in VPA-treated rats.

### 3.5 OT attenuates disruption of the BBB and inhibits TLR-4/IκB-α/NF-κB signaling pathway in the cortex of VPA-treated rats

**Figure 5:**
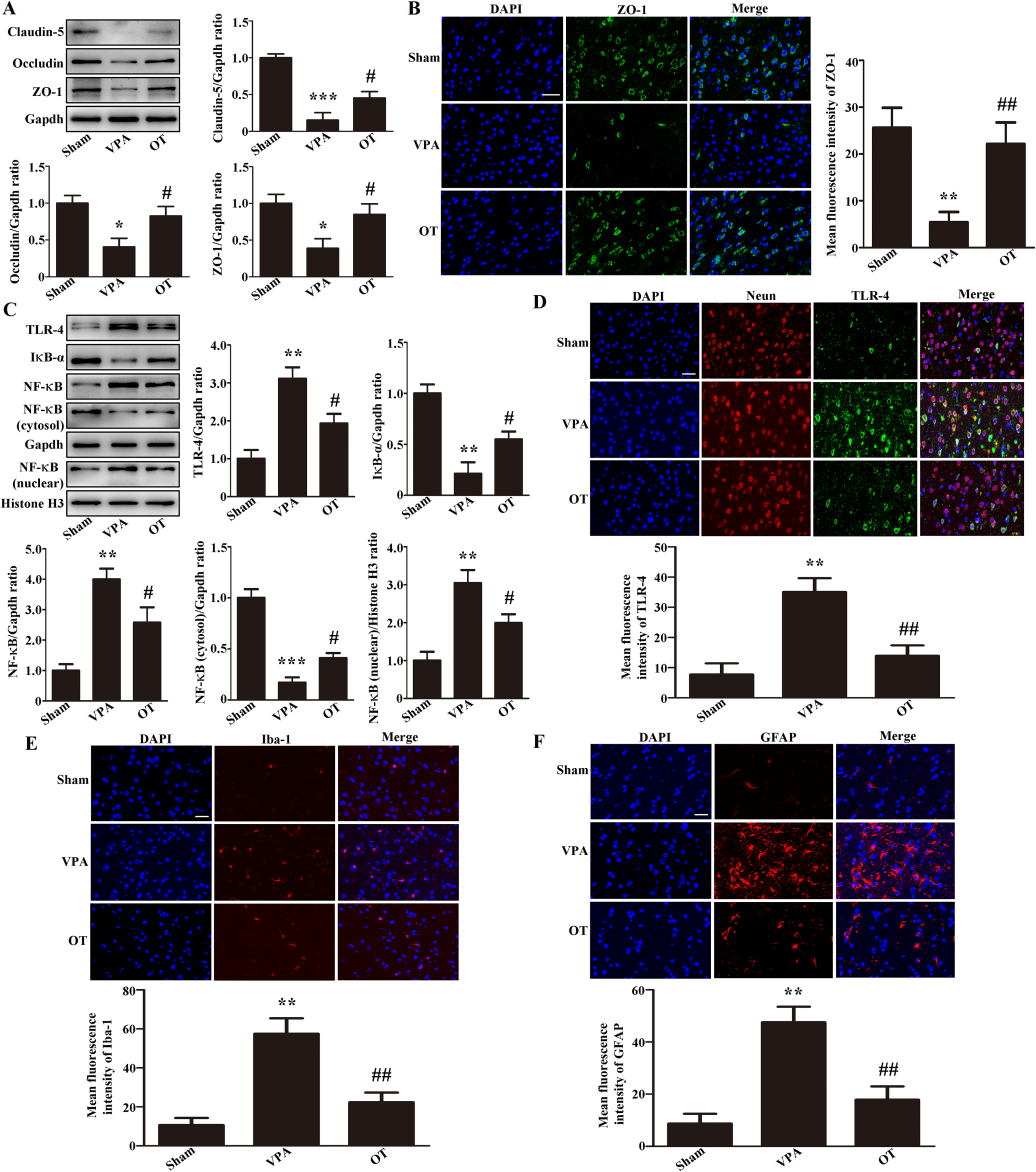
OT attenuates BBB disruption and inhibits the TLR-4/IκB-α/NF-κB signaling pathway in the cortex of VPA-treated rats. **(A)** Representative images and quantification of claudin-5, occludin, and ZO-1 expression in the cortex. The levels were normalized to GAPDH. **(B)** Representative images and quantification of ZO-1-positive cells in the cortex. **(C)** Representative images and quantification of TLR-4, IκB-α, NF-κB, NF-κB (cytosol), and NF-κB (nuclear) expression in the cortex. The levels of TLR-4, IκB-α, NF-κB, and NF-κB (cytosol) were normalized to GAPDH. The level of NF-κB (nuclear) was normalized to Histone H3. **(D)** Representative images and quantification of TLR-4-positive neurons in the cortex. **(E)** Representative images and quantification of Iba-1-positive cells in the cortex. **(F)** Representative images and quantification of GFAP-positive cells in the cortex. The data are expressed as the mean ± SEM. *n* = 3/group. ^*^p < 0.05, ^**^p < 0.01, and ^***^p < 0.001 for the VPA group versus the sham group; ^#^*p* < 0.05, and ^##^*p* < 0.01 for the OT group versus the VPA group.

To evaluate whether OT affects BBB function, Western blotting was used to analyze tight junction proteins in the cortex. In comparison to the sham group, the VPA group exhibited notably reduced expression of claudin-5, occludin, and ZO-1 in the cortex (Figure 5A, *p* < 0.05 or *p* < 0.001). In contrast, the OT group showed increased expression of these proteins in the cortex relative to the VPA group (Figure 5A, *p* < 0.05). Immunofluorescence staining further revealed that the OT group had more ZO-1-positive cells in the cortex than the VPA group (Figure 5B, *p* < 0.01).

We next examined whether OT affected the TLR-4/IκB-α/NF-κB signaling pathway in the cortex of VPA-treated rats. In comparison to the sham group, the VPA group exhibited upregulated expression of TLR-4, NF-κB, and NF-κB (nuclear) in the cortex, with significant downregulation of IκB-α and NF-κB (cytosol) (Figure 5C, *p* < 0.01 or *p* < 0.001). In contrast, the OT group exhibited downregulated TLR-4, NF-κB, and NF-κB (nuclear) expression, accompanied by upregulated IκB-α and NF-κB (cytosol) expression, compared to the VPA group (Figure 5C, *p* < 0.05). Furthermore, we performed multiplex immunofluorescence staining on the cortex. TLR-4 was co-stained with Neun, Iba-1, and GFAP, respectively. We found that TLR-4 was co-expressed with Neun, but not with Iba-1 and GFAP. The inhibition of the TLR-4 pathway by OT mainly occurs in neurons. Immunofluorescence staining further revealed that the OT group had fewer TLR-4-positive neurons in the cortex than the VPA group (Figure 5D, *p* < 0.01). These data imply that OT attenuates BBB disruption and inhibits the TLR-4/IκB-α/NF-κB signaling pathway.

We next examined whether OT attenuated neuroinflammation in the cortex of VPA-treated rats. The VPA group had more Iba-1-positive cells and GFAP-positive cells than the sham group (Figures 5E,F, *p* < 0.01). In contrast, the OT group demonstrated significantly reduced expression of Iba-1-positive cells and GFAP-positive cells relative to the VPA group (Figures 5E,F, *p* < 0.01). These findings imply that OT attenuated neuroinflammation in the cortex of VPA-treated rats.

### 3.6 Antibiotics eliminated the neuroprotective effects of OT by interfering with gut microbiota in VPA-treated rats

**Figure 6:**
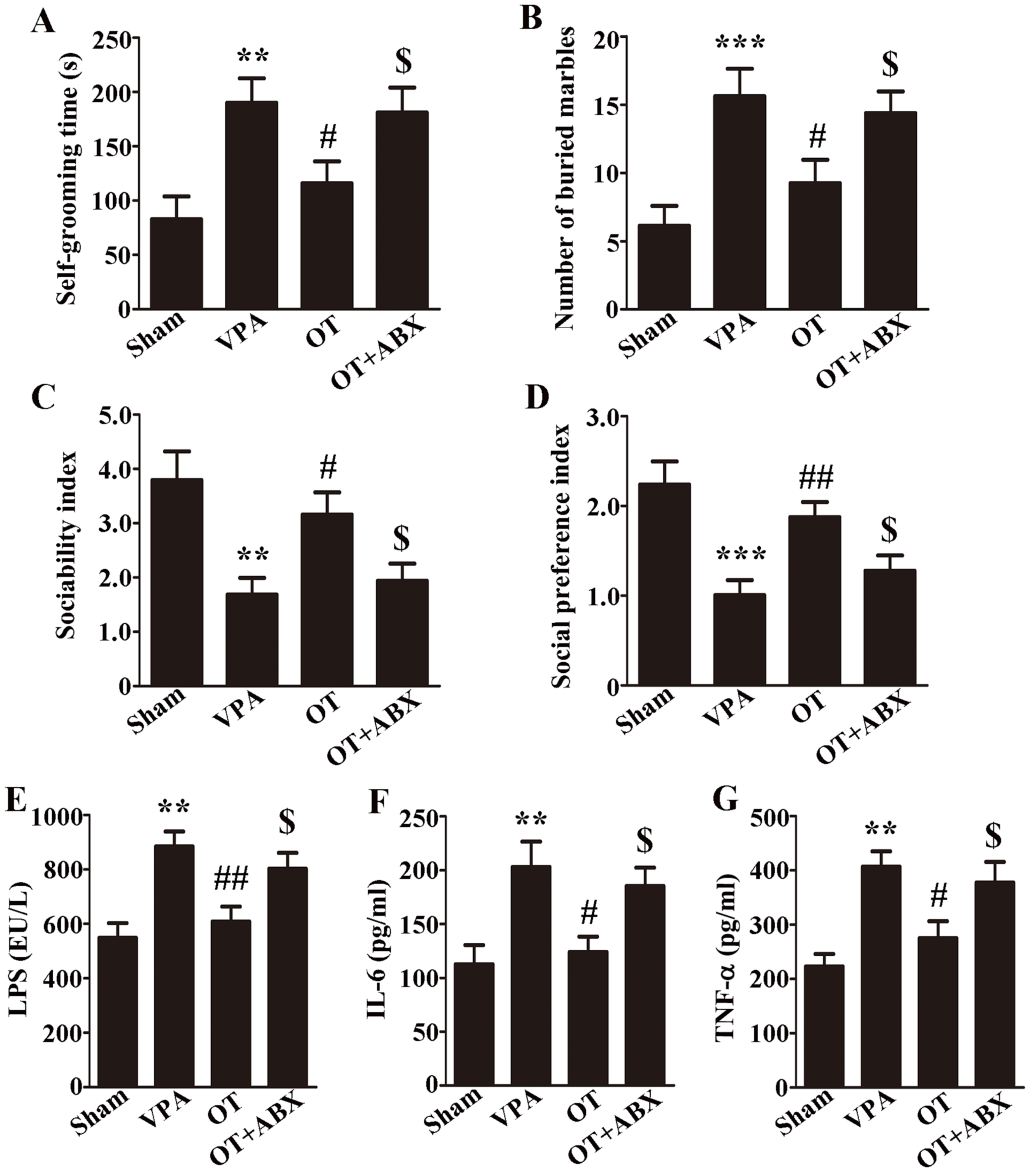
Antibiotics eliminate the neuroprotective effects of OT in VPA-treated rats. **(A)** Self-grooming time; *n* = 6/group. **(B)** Number of marbles buried in the marble burying test; *n* = 8/group. **(C)** Sociability index; *n* = 6/group. **(D)** Social preference index; *n* = 6/group. **(E–G)** Cortical levels of LPS, IL-6, and TNF-α; *n* = 3/group. The data are expressed as the mean ± SEM. ^**^p < 0.01, and ^***^p < 0.001 for the VPA group versus the sham group; ^#^*p* < 0.05, and ^##^*p* < 0.01 for the OT group versus the VPA group; ^$^*p* < 0.05 for the OT + ABX group versus the OT group.

To obtain direct evidence of the neuroprotective effect of OT on gut microbiota, we further administered an antibiotic cocktail (ABX) treatment to deplete gut microbiota, after which behavioral tests were conducted and levels of LPS and proinflammatory cytokines in the cortex were reassessed. In comparison with the OT group, the antibiotic treatment markedly increased self-grooming time and the number of buried marbles and reduced both the sociability and social preference indices in VPA-treated rats (Figures 6A–D, *p* < 0.05). Additionally, ELISA revealed that in comparison with the OT group, the OT + ABX group exhibited significantly elevated levels of LPS (Figure 6E, *p* < 0.05), IL-6 (Figure 6F, *p* < 0.05), and TNF-α (Figure 6G, *p* < 0.05) in the cortex. In summary, antibiotic treatment eliminated the neuroprotective effects of OT and increased neuroinflammation in VPA-treated rats. These findings indicate that gut microbiota plays an important role in the neuroprotection of OT in VPA-treated rats.

## 4 Discussion

Our study provides the first experimental evidence that OT attenuates neuroinflammation in a VPA-induced autism rat model by modulating the microbiota-gut-brain axis through inhibition of TLR-4/IκB-α/NF-κB signaling pathway. These findings advance our understanding of dietary interventions for ASD by elucidating a multi-organ protective mechanism involving gut microbiota remodeling, barrier restoration, and neuronal TLR-4 suppression.

The microbiota-gut-brain axis constitutes a critical bidirectional communication network between the gastrointestinal tract and the central nervous system, profoundly influencing brain development, function, and behavior (5, 6, 41). Dysregulation of this axis, often characterized by gut dysbiosis and compromised barrier integrity, facilitates the translocation of microbial products like LPS into systemic circulation and potentially across the BBB, thereby contributing to neuroinflammation and neurological disorders, including ASD (42). Consistent with clinical observations (7, 8, 43–45), our VPA rat model exhibited significant gut dysbiosis, marked by increased abundances of *Ruminococcaceae* (family), *Bacteroides* (genus), and *Ruminococcus* (genus). This dysbiosis aligns with emerging evidence highlighting VPA’s impact beyond direct neuronal HDAC inhibition, specifically its disruption of gut microbiota homeostasis, downregulation of intestinal tight junction proteins (21), increased intestinal permeability, and promotion of systemic LPS translocation (22). Consequently, we observed elevated levels of LPS and proinflammatory cytokines (IL-6, TNF-α) in the plasma, intestine, and cortex, alongside impaired integrity of both the intestinal barrier and BBB. OT treatment effectively reversed these VPA-induced perturbations, restoring barrier function and reducing systemic and neuroinflammatory markers.

The observed dysbiosis in our model, particularly the enrichment of *Bacteroides* [known for LPS production and association with inflammation (46)] and *Ruminococcus* [which, while important for short-chain fatty acids production, can exacerbate inflammation and barrier damage under dysbiotic conditions (47–49)], underscores a clear link between gut microbial shifts and ASD-like pathophysiology. Modulation of gut microbiota represents a promising therapeutic avenue for neuropsychiatric conditions (50, 51). OT, recognized for its neuroprotective potential in other disorders like Parkinson’s and Alzheimer’s disease (12, 52), significantly ameliorated the core behavioral deficits in VPA-treated rats. Specifically, the high dose of OT (400 mg/kg/day) reduced repetitive behaviors (self-grooming test and marble burying test) and improved sociability in a dose-dependent manner, with lower doses (100, 200 mg/kg/day) showing non-significant trends. Furthermore, OT attenuated neuronal damage in the cortex. Crucially, 16S rRNA analysis revealed that OT treatment normalized the VPA-induced dysbiosis, significantly reducing the elevated abundances of *Ruminococcaceae*, *Bacteroides*, and *Ruminococcus*. This modulation of detrimental microbial populations likely underpins the subsequent reduction in inflammation.

The reduction in gut-derived LPS is pivotal. LPS activates TLR-4, which triggers the degradation of IκB-α and nuclear translocation of NF-κB, leading to the transcription of proinflammatory cytokines (26, 27). TLR-4/NF-κB pathway activation is implicated in neuroinflammation across various neurological disorders (53, 54). Our data show that VPA exposure activated the TLR-4/IκB-α/NF-κB pathway in both the intestine and cortex. OT supplementation potently inhibited this pathway, as evidenced by decreased TLR-4 and nuclear NF-κB expression and increased IκB-α and cytosolic NF-κB expression in both tissues. Importantly, multiplex immunofluorescence revealed that cortical TLR-4 expression was predominantly neuronal (co-localized with Neun, not Iba-1 or GFAP), suggesting that neurons are a primary site for OT’s modulation of this pathway within the brain. Furthermore, OT significantly reduced the activation of microglia (Iba-1+) and astrocytes (GFAP+), which are key cellular mediators of neuroinflammation (55). This provides direct evidence of attenuated neuroinflammatory responses downstream of neuronal TLR-4/NF-κB signaling. This cascade—from reduced LPS burden due to improved barrier function and microbial modulation to inhibition of neuronal TLR-4 signaling and consequent dampening of glial activation—forms the core mechanism by which OT exerts its neuroprotective effects.

**Figure 7:**
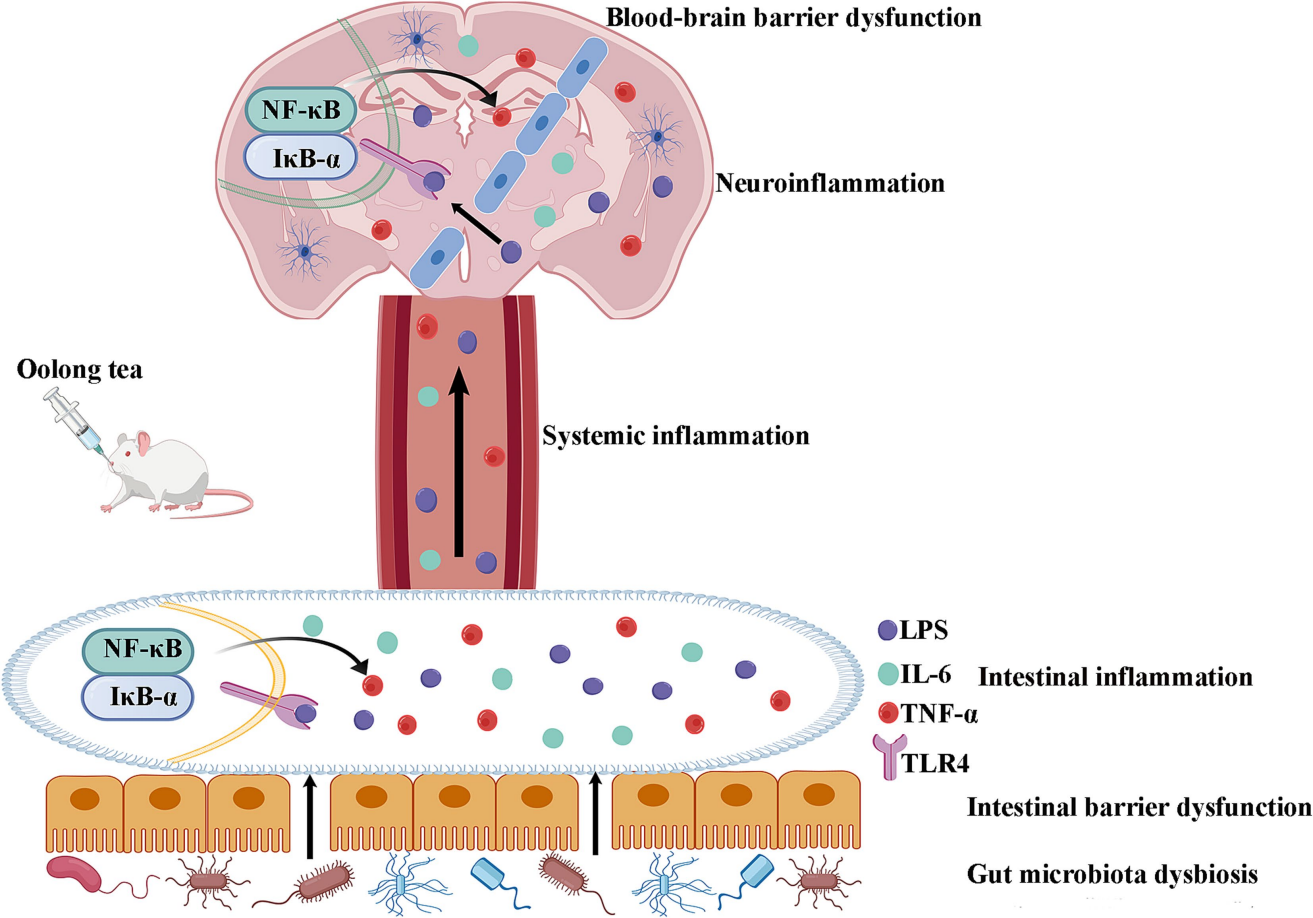
Schematic diagram of the mechanism of OT through modulation of the microbiota-gut-brain axis by suppressing TLR-4/IκB-α/NF-κB signaling pathway in a rat model of autism. OT reduces intestinal permeability and suppresses intestinal inflammation by regulating the gut microbiota imbalance in VPA-treated rats. The reduction in intestinal permeability prevents microbial toxins and proinflammatory cytokines from penetrating the circulation and inhibiting systemic inflammation. OT attenuates the BBB, inhibits neuroinflammation, and exerts a protective effect on the brain. Further mechanistic studies find that OT exerts its protective effect by suppressing the TLR-4/IκB-α/NF-κB signaling pathway in the intestines and brains.

The critical dependence of OT’s neuroprotection on gut microbiota was unequivocally demonstrated by the antibiotic depletion experiment. Depleting the gut microbiota with ABX abolished the behavioral improvements and anti-neuroinflammatory effects conferred by OT, reinstating elevated levels of cortical LPS, IL-6, and TNF-α. This finding provides direct evidence that the beneficial effects of OT are mediated primarily through its interaction with the gut microbial community. While OT contains bioactive components like caffeine, theanine, and catechins with known anti-inflammatory properties, our results indicate that their neuroprotective efficacy in this ASD model is fundamentally dependent on the modulation of the gut microbiota. OT ameliorates intestinal dysbiosis, reduces LPS leakage, dampens systemic inflammation, preserves intestinal and BBB integrity, and ultimately suppresses neuroinflammation through the inhibition of the neuronal TLR-4/IκB-α/NF-κB pathway (Figure 7).

Despite providing compelling evidence for OT’s modulation of the microbiota-gut-brain axis in ameliorating ASD-like phenotypes, this study has several limitations. First, the absence of fecal microbiota transplantation (FMT) experiments prevents us from establishing a definitive causal link between the specific microbial shifts induced by OT and the observed behavioral improvements. Second, the lack of a TLR-4 inhibitor control group makes it difficult to conclusively dissect the contribution of TLR-4 pathway inhibition from other potential effects of OT’s bioactive components. Third, the analysis of OT metabolites, particularly microbial-derived phenolic acids potentially mediating systemic effects, was insufficient. Finally, fecal sampling at only a single postnatal timepoint (PND49) limits our understanding of the developmental dynamics of microbial colonization and OT’s impact thereon. Addressing these limitations will be a focus of future research, incorporating longitudinal sampling across key developmental stages (e.g., PND21 to PND49) and comprehensive metabolomic profiling of catechin derivatives and microbial metabolites.

Although based on an animal model, the translational potential of these findings is supported by several lines of evidence. The OT dose used (400 mg/kg/day) translates to approximately 28 g of dry tea leaves per day for a 70-kg human, which falls within the safe consumption range established by the WHO. The shared gut microbiota features (e.g., increased *Bacteroides*) between VPA-exposed rats and children with ASD (43, 44) strengthen the model’s relevance. Critically, the success of microbiota-targeted therapies, including FMT, in improving gastrointestinal and behavioral symptoms in ASD clinical trials validates the therapeutic approach targeting the gut-brain axis (56, 57). Future investigations should prioritize examining potential sex differences in OT’s effects, given their importance in ASD (44), which were not addressed in this study. Additionally, assessing the long-term impact of OT on cognitive development is crucial. In conclusion, by targeting the “microbiota-barrier-TLR4” axis, OT offers a novel adjunctive therapeutic strategy for ASD, particularly relevant given the high prevalence of gastrointestinal symptoms [affecting 9–91% of ASD children (58)], while potentially circumventing some risks associated with invasive procedures like FMT.

## 5 Conclusion

OT attenuates ASD-like phenotypes and neuroinflammation in VPA-exposed rats by targeting the microbiota-gut-brain axis. It ameliorates core behavioral deficits, restores gut microbiota composition (notably reducing *Ruminococcaceae* and *Bacteroides*), and enhances intestinal and BBB by upregulating tight junction proteins. Critically, OT suppresses systemic and neuroinflammation by inhibiting the TLR-4/IκB-α/NF-κB signaling pathway, with TLR-4 predominantly localized to cortical neurons. The abolition of OT’s neuroprotective effects by antibiotic-induced microbiota depletion confirms gut microbiota dependency. This study establishes OT as a microbiota-modulating intervention that mitigates ASD pathophysiology through multi-organ barrier restoration and neuronal TLR-4 pathway inhibition, offering a novel dietary strategy for ASD management.

## Data Availability

The raw data supporting the conclusions of this article will be made available by the authors, without undue reservation.

## Footnotes

^1^ https://ftp.arb-silva.de

## References

[1] Maenner MJ Warren Z Williams AR Amoakohene E Bakian AV Bilder DA. Prevalence and characteristics of autism spectrum disorder among children aged 8 years—autism and developmental disabilities monitoring network, 11 sites, United States, 2020. MMWR Surveill Summ. (2023) 72:1–14. doi: 10.15585/mmwr.ss7202a1, PMID: 36952288 PMC10042614

[2] Zhang P Liu Y Jin X Hu Z Yang J Lu H. Alzheimer’s disease-like pathology induced by *Porphyromonas gingivalis* in middle-aged mice is mediated by NLRP3 inflammasome via the microbiota-gut-brain axis. J Alzheimer’s Dis. (2025) 103:487–505. doi: 10.1177/13872877241302498, PMID: 39639573

[3] Gao W Wu X Wang Y Lu F Liu F. Brazilin-rich extract from *Caesalpinia sappan* L. attenuated the motor deficits and neurodegeneration in MPTP/p-induced Parkinson's disease mice by regulating gut microbiota and inhibiting inflammatory responses. ACS Chem Neurosci. (2025) 16:181–94. doi: 10.1021/acschemneuro.4c00679, PMID: 39711007

[4] Cheng L Wu H Cai X Zhang Y Yu S Hou Y. A Gpr35-tuned gut microbe-brain metabolic axis regulates depressive-like behavior. Cell Host Microbe. (2024) 32:227–243.e6. doi: 10.1016/j.chom.2023.12.009, PMID: 38198925

[5] Needham BD Funabashi M Adame MD Wang Z Boktor JC Haney J. A gut-derived metabolite alters brain activity and anxiety behaviour in mice. Nature. (2022) 602:647–53. doi: 10.1038/s41586-022-04396-8, PMID: 35165440 PMC9170029

[6] Cui S Aronno M Wong AKQ Snodgrass L. The overlooked role of microbiota-gut-brain communication in child psychiatry: a call for integration in early intervention strategies. Commun Integr Biol. (2025) 18:2446332. doi: 10.1080/19420889.2024.2446332 39764483 PMC11702936

[7] Lou M Cao A Jin C Mi K Xiong X Zeng Z. Deviated and early unsustainable stunted development of gut microbiota in children with autism spectrum disorder. Gut. (2022) 71:1588–99. doi: 10.1136/gutjnl-2021-325115, PMID: 34930815 PMC9279844

[8] Wan Y Zuo T Xu Z Zhang F Zhan H Chan D. Underdevelopment of the gut microbiota and bacteria species as non-invasive markers of prediction in children with autism spectrum disorder. Gut. (2022) 71:910–8. doi: 10.1136/gutjnl-2020-324015, PMID: 34312160

[9] Prince N Peralta Marzal LN Roussin L Monnoye M Philippe C Maximin E. Mouse strain-specific responses along the gut-brain axis upon fecal microbiota transplantation from children with autism. Gut Microbes. (2025) 17:2447822. doi: 10.1080/19490976.2024.2447822, PMID: 39773319 PMC11730631

[10] Wu J Deng X Sun Y Li J Dai H Qi S. Aged oolong tea alleviates dextran sulfate sodium-induced colitis in mice by modulating the gut microbiota and its metabolites. Food Chem X. (2024) 21:101102. doi: 10.1016/j.fochx.2023.101102, PMID: 38268839 PMC10805651

[11] Zhang X Meng X Liu Y Yang X Chen J Liu T. Roles of oolong tea extracts in the protection against *Staphylococcus aureus* infection in *Caenorhabditis elegans*. J Food Sci. (2025) 90:e17651. doi: 10.1111/1750-3841.17651, PMID: 39801228

[12] Jhuo CF Chen CJ Tzen JTC Chen WY. Teaghrelin protected dopaminergic neurons in MPTP-induced Parkinson’s disease animal model by promoting PINK1/Parkin-mediated mitophagy and AMPK/SIRT1/PGC1-α-mediated mitochondrial biogenesis. Environ Toxicol. (2024) 39:4022–34. doi: 10.1002/tox.24275, PMID: 38622810

[13] Song Z Ho CT Zhang X. Gut microbiota mediate the neuroprotective effect of oolong tea polyphenols in cognitive impairment induced by circadian rhythm disorder. J Agric Food Chem. (2024) 72:12184–97. doi: 10.1021/acs.jafc.4c01922, PMID: 38745351

[14] Miao C Shen Y Lang Y Li H Gong Y Liu Y. Biomimetic nanoparticles with enhanced rapamycin delivery for autism spectrum disorder treatment via autophagy activation and oxidative stress modulation. Theranostics. (2024) 14:4375–92. doi: 10.7150/thno.95614, PMID: 39113803 PMC11303075

[15] Meng J Pan P Guo G Chen A Meng X Liu H. Transient CSF1R inhibition ameliorates behavioral deficits in *Cntnap2* knockout and valproic acid-exposed mouse models of autism. J Neuroinflammation. (2024) 21:262. doi: 10.1186/s12974-024-03259-5, PMID: 39425203 PMC11487716

[16] Chanda S Ang CE Lee QY Ghebrial M Haag D Shibuya Y. Direct reprogramming of human neurons identifies MARCKSL1 as a pathogenic mediator of valproic acid-induced teratogenicity. Cell Stem Cell. (2019) 25:103–119.e6. doi: 10.1016/j.stem.2019.04.021, PMID: 31155484 PMC6609489

[17] Li S Zhang N Li W Zhang HL Wang XX. Gastrointestinal problems in a valproic acid-induced rat model of autism: from maternal intestinal health to offspring intestinal function. World J Psychiatry. (2024) 14:1095–105. doi: 10.5498/wjp.v14.i7.1095, PMID: 39050201 PMC11262932

[18] Li B Xiong Y Li Y. The impact of valproic acid on microbiota in a mouse model of autism spectrum disorder. Psychiatry Clin Psychopharmacol. (2025) 35:6–13. doi: 10.5152/pcp.2025.24966, PMID: 40224943 PMC11992932

[19] Liu S Xi H Xue X Sun X Huang H Fu D. *Clostridium butyricum* regulates intestinal barrier function via trek1 to improve behavioral abnormalities in mice with autism spectrum disorder. Cell Biosci. (2024) 14:95. doi: 10.1186/s13578-024-01278-6 39034406 PMC11265103

[20] Prince N Peralta Marzal LN Markidi A Ahmed S Adolfs Y Pasterkamp RJ. Prebiotic diet normalizes aberrant immune and behavioral phenotypes in a mouse model of autism spectrum disorder. Acta Pharmacol Sin. (2024) 45:1591–603. doi: 10.1038/s41401-024-01268-x 38589690 PMC11272935

[21] Yang X Li H Yang C Ge J. Supplementation with stigma maydis polysaccharide attenuates autism-like behaviors and improves gut function in valproic acid-induced autism model male rats. Int J Dev Neurosci. (2024) 84:567–80. doi: 10.1002/jdn.10354, PMID: 38923604

[22] Palanivelu L Chen YY Liang YW Li SJ Chang CW Huang YT. Diffusion kurtosis imaging biomarkers associated with amelioration of neuroinflammation, gray matter microstructural abnormalities, and gut dysbiosis by central thalamic deep brain stimulation in autistic-like young rats. NeuroImage. (2025) 317:121344. doi: 10.1016/j.neuroimage.2025.121344 40544899

[23] Fakir S Sarker MMR Sigdel M Barabutis N. Protective effects of Pasireotide in LPS-induced acute lung injury. Pharmaceuticals. (2025) 18:942. doi: 10.3390/ph18070942, PMID: 40732232 PMC12298338

[24] Wang X Hu R Lin F Yang T Lu Y Sun Z. *Lactobacillus reuteri* or *Lactobacillus rhamnosus* GG intervention facilitates gut barrier function, decreases corticosterone and ameliorates social behavior in LPS-exposed offspring. Food Res Int. (2024) 197:115212. doi: 10.1016/j.foodres.2024.115212, PMID: 39593298

[25] Xiao L Yan J Yang T Zhu J Li T Wei H. Fecal microbiome transplantation from children with autism spectrum disorder modulates tryptophan and serotonergic synapse metabolism and induces altered behaviors in Germ-free mice. mSystems. (2021) 6:e01343-20. doi: 10.1128/mSystems.01343-20, PMID: 33824200 PMC8547010

[26] Yilmaz DE Senol SP Temiz-Resitoglu M Sahan-Firat S Tunctan B. NLRX1 ligand, docosahexaenoic acid, ameliorates LPS-induced inflammatory hyperalgesia by decreasing TRAF6/IKK/IκB-a/NF-κB signaling pathway activity. Cell Mol Biol. (2023) 69:15–23. doi: 10.14715/cmb/2023.69.9.3, PMID: 37807339

[27] Shyni GL Renjitha J Somappa SB Raghu KG. Zerumin a attenuates the inflammatory responses in LPS-stimulated H9c2 cardiomyoblasts. J Biochem Mol Toxicol. (2021) 35:1–11. doi: 10.1002/jbt.22777, PMID: 33755281

[28] Gurram PC Manandhar S Satarker S Mudgal J Arora D Nampoothiri M. Dopaminergic signaling as a plausible modulator of astrocytic toll-like receptor 4: a crosstalk between neuroinflammation and cognition. CNS Neurol Disord Drug Targets. (2023) 22:539–57. doi: 10.2174/1871527321666220413090541, PMID: 35422229

[29] Qiu Z Liao J Chen J Li A Lin M Liu H. Comprehensive analysis of fresh tea (*Camellia sinensis* cv. Lingtou Dancong) leaf quality under different nitrogen fertilization regimes. Food Chem. (2024) 439:138127. doi: 10.1016/j.foodchem.2023.138127, PMID: 38064834

[30] Banji D Banji OJ Abbagoni S Hayath MS Kambam S Chiluka VL. Amelioration of behavioral aberrations and oxidative markers by green tea extract in valproate induced autism in animals. Brain Res. (2011) 1410:141–51. doi: 10.1016/j.brainres.2011.06.063, PMID: 21820650

[31] Sun L Xu H Ye J Gaikwad NW. Comparative effect of black, green, oolong, and white tea intake on weight gain and bile acid metabolism. Int Nutrition. (2019) 65:208–15. doi: 10.1016/j.nut.2019.02.006, PMID: 31031064

[32] Schimidt HL Carrazoni GS Garcia A Izquierdo I Mello-Carpes PB Carpes FP. Strength training or green tea prevent memory deficits in a β-amyloid peptide-mediated Alzheimer’s disease model. Exp Gerontol. (2021) 143:111186. doi: 10.1016/j.exger.2020.111186, PMID: 33279659

[33] Shi H Yu Y Lin D Zheng P Zhang P Hu M. β-glucan attenuates cognitive impairment via the gut-brain axis in diet-induced obese mice. Microbiome. (2020) 8:143. doi: 10.1186/s40168-020-00920-y, PMID: 33008466 PMC7532656

[34] Cai H Zhang C Zhang H Du Y Wang K. Deletion of Mex3c gene leads to autistic-like behavior in mice by inhibiting AMPK signal pathway. Front Behav Neurosci. (2025) 19:1551440. doi: 10.3389/fnbeh.2025.1551440, PMID: 40463420 PMC12129987

[35] Kalueff AV Stewart AM Song C Berridge KC Graybiel AM Fentress JC. Neurobiology of rodent self-grooming and its value for translational neuroscience. Nat Rev Neurosci. (2016) 17:45–59. doi: 10.1038/nrn.2015.8, PMID: 26675822 PMC4840777

[36] Jayaprakash P Isaev D Yang KS Beiram R Oz M Sadek B. Apigenin alleviates autistic-like stereotyped repetitive behaviors and mitigates brain oxidative stress in mice. Pharmaceuticals. (2024) 17:482. doi: 10.3390/ph17040482 38675442 PMC11054933

[37] Wang Y Wang Y Tang J Li R Jia Y Yang H. Impaired neural circuitry of hippocampus in Pax2 nervous system-specific knockout mice leads to restricted repetitive behaviors. CNS Neurosci Ther. (2024) 30:e14482. doi: 10.1111/cns.14482, PMID: 37786962 PMC11017408

[38] Liu L Zhou X Ma Z Liu R Zhang Y Wang Y. Hippocampal proteomics reveals the novel molecular profiling of postnatal lead (Pb) exposure on autism-like behaviors. Toxics. (2025) 13:465. doi: 10.3390/toxics13060465, PMID: 40559938 PMC12197489

[39] Segata N Izard J Waldron L Gevers D Miropolsky L Garrett WS. Metagenomic biomarker discovery and explanation. Genome Biol. (2011) 12:R60. doi: 10.1186/gb-2011-12-6-r60 21702898 PMC3218848

[40] Zheng Z Zhang L Qu Y Xiao G Li S Bao S. Mesenchymal stem cells protect against hypoxia-ischemia brain damage by enhancing autophagy through brain derived neurotrophic factor/mammalin target of rapamycin signaling pathway. Stem Cells. (2018) 36:1109–21. doi: 10.1002/stem.2808 29451335 PMC6657778

[41] Aburto MR Cryan JF. Gastrointestinal and brain barriers: unlocking gates of communication across the microbiota-gut-brain axis. Nat Rev Gastroenterol Hepatol. (2024) 21:222–47. doi: 10.1038/s41575-023-00890-0, PMID: 38355758

[42] Yuan X Ouedraogo SY Jammeh ML Simbiliyabo L Jabang JN Jaw M. Can microbiota gut-brain axis reverse neurodegenerative disorders in human? Ageing Res Rev. (2025) 104:102664. doi: 10.1016/j.arr.2025.102664, PMID: 39818235

[43] Chen YC Lin HY Chien Y Tung YH Ni YH Gau SS. Altered gut microbiota correlates with behavioral problems but not gastrointestinal symptoms in individuals with autism. Brain Behav Immun. (2022) 106:161–78. doi: 10.1016/j.bbi.2022.08.015, PMID: 36058421

[44] Carmel J Ghanayem N Mayouf R Saleev N Chaterjee I Getselter D. Bacteroides is increased in an autism cohort and induces autism-relevant behavioral changes in mice in a sex-dependent manner. npj Biofilms Microbiomes. (2023) 9:103. doi: 10.1038/s41522-023-00469-2, PMID: 38110423 PMC10728123

[45] David MM Tataru C Daniels J Schwartz J Keating J Hampton-Marcell J. Children with autism and their typically developing siblings differ in amplicon sequence variants and predicted functions of stool-associated microbes. mSystems. (2021) 6:e00193-20. doi: 10.1128/mSystems.00193-20, PMID: 33824194 PMC8561662

[46] Yan Y Zhou X Guo K Zhou F Yang H. Chlorogenic acid protects against indomethacin-induced inflammation and mucosa damage by decreasing bacteroides-derived LPS. Front Immunol. (2020) 11:1125. doi: 10.3389/fimmu.2020.01125, PMID: 32582202 PMC7283755

[47] Li N Wang H Pei H Wu Y Li L Ren Y. Genus_Ruminococcus and order_Burkholderiales affect osteoporosis by regulating the microbiota-gut-bone axis. Front Microbiol. (2024) 15:1373013. doi: 10.3389/fmicb.2024.1373013, PMID: 38835486 PMC11148449

[48] Han Y Quan X Chuang Y Liang Q Li Y Yuan Z. A multi-omics analysis for the prediction of neurocognitive disorders risk among the elderly in Macao. Clin Transl Med. (2022) 12:e909. doi: 10.1002/ctm2.909, PMID: 35696554 PMC9191869

[49] Zhai L Huang C Ning Z Zhang Y Zhuang M Yang W. *Ruminococcus gnavus* plays a pathogenic role in diarrhea-predominant irritable bowel syndrome by increasing serotonin biosynthesis. Cell Host Microbe. (2023) 31:33–44.e5. doi: 10.1016/j.chom.2022.11.006, PMID: 36495868

[50] Wang Z Wang ZX Xu KF An Y Cui M Zhang X. A metal-polyphenol-based antidepressant for alleviating colitis-associated mental disorders. Adv Mater. (2025) 37:e2410993. doi: 10.1002/adma.202410993, PMID: 39623787

[51] Zhong D Jin K Wang R Chen B Zhang J Ren C. Microalgae-based hydrogel for inflammatory bowel disease and its associated anxiety and depression. Adv Mater. (2024) 36:e2312275. doi: 10.1002/adma.202312275, PMID: 38277492

[52] Tu Y Kang H Kim E Yang J He P Wu Y. Metabolomics study suggests the mechanism of different types of tieguanyin (oolong) tea in alleviating Alzheimer’s disease in APP/PS1 transgenic mice. Metabolites. (2022) 12:466. doi: 10.3390/metabo12050466, PMID: 35629970 PMC9142883

[53] da Silva RS de Paiva IHR Mendonça IP de Souza JRB Lucena-Silva N Peixoto CA. Anorexigenic and anti-inflammatory signaling pathways of semaglutide via the microbiota-gut-brain axis in obese mice. Inflammopharmacology. (2025) 33:845–64. doi: 10.1007/s10787-024-01603-y, PMID: 39586940

[54] Wu W Xu L Mu D Wang D Tan S Liu L. Ethanol extracts of *Cinnamomum migao* H.W. Li attenuates neuroinflammation in cerebral ischemia-reperfusion injury via regulating TLR4-PI3K-Akt-NF-κB pathways. J Ethnopharmacol. (2025) 339:119150. doi: 10.1016/j.jep.2024.119150, PMID: 39580135

[55] Yuan H Lu B Sun D Chen J Fang X. CCL2 inhibitor bindarit improve postoperative cognitive function by attenuating pericyte loss-related blood-brain barrier disruption and neuroinflammation. Mediat Inflamm. (2025) 2025:7248780. doi: 10.1155/mi/7248780 PMC12178779 40548298

[56] Liber A Więch M. The impact of fecal microbiota transplantation on gastrointestinal and behavioral symptoms in children and adolescents with autism spectrum disorder: a systematic review. Nutrients. (2025) 17:2250. doi: 10.3390/nu17132250 40647353 PMC12252074

[57] Kang DW Adams JB Gregory AC Borody T Chittick L Fasano A. Microbiota transfer therapy alters gut ecosystem and improves gastrointestinal and autism symptoms: an open-label study. Microbiome. (2017) 5:10. doi: 10.1186/s40168-016-0225-7, PMID: 28122648 PMC5264285

[58] Leader G Abberton C Cunningham S Gilmartin K Grudzien M Higgins E. Gastrointestinal symptoms in autism spectrum disorder: a systematic review. Nutrients. (2022) 14:1471. doi: 10.3390/nu14071471 35406084 PMC9003052

